# Modifying the DPClus algorithm for identifying protein complexes based on new topological structures

**DOI:** 10.1186/1471-2105-9-398

**Published:** 2008-09-25

**Authors:** Min Li, Jian-er Chen, Jian-xin Wang, Bin Hu, Gang Chen

**Affiliations:** 1 School of Information Science and Engineering, Central South University, Changsha, Hunan 410083, PR China; 2 Department of Computer Science, Texas A&M University, College Station, Texas 77843, USA

## Abstract

**Background:**

Identification of protein complexes is crucial for understanding principles of cellular organization and functions. As the size of protein-protein interaction set increases, a general trend is to represent the interactions as a network and to develop effective algorithms to detect significant complexes in such networks.

**Results:**

Based on the study of known complexes in protein networks, this paper proposes a new topological structure for protein complexes, which is a combination of subgraph diameter (or average vertex distance) and subgraph density. Following the approach of that of the previously proposed clustering algorithm DPClus which expands clusters starting from seeded vertices, we present a clustering algorithm IPCA based on the new topological structure for identifying complexes in large protein interaction networks. The algorithm IPCA is applied to the protein interaction network of Sacchromyces cerevisiae and identifies many well known complexes. Experimental results show that the algorithm IPCA recalls more known complexes than previously proposed clustering algorithms, including DPClus, CFinder, LCMA, MCODE, RNSC and STM.

**Conclusion:**

The proposed algorithm based on the new topological structure makes it possible to identify dense subgraphs in protein interaction networks, many of which correspond to known protein complexes. The algorithm is robust to the known high rate of false positives and false negatives in data from high-throughout interaction techniques. The program is available at .

## Background

In the post-genomic era, one of the most important issues is to systematically analyze and comprehensively understand the topology of biological networks and biochemical progress in cells. Protein complexes can help us to understand certain biological progress and to predict the functions of proteins. As John Donne pointed out, no protein is an island entire of itself or at least, very few proteins are. Most proteins seem to function within complicated cellular pathways, interacting with other proteins either in pairs or as components of larger complexes [1,2].

Various methods have been used to detect protein complexes. Large-scale mass-spectrometric studies in Saccharomyces cerevisiae provide a compendium of protein complexes that are considered to play a key role in carrying out yeast functionality [3,4]. Although vastly informative, such methods offer information only on the composition of a protein complex at a given time and developmental or environmental condition [5]. Repeated individual purifications coupled with each of these complexes could offer a more precise picture [6,7], but such approaches on a large scale are unavailable at present. Affinity purification techniques using mass spectrometry provide a particularly effective approach to identifying protein complexes [1]. These high-throughput techniques have been used to perform large scale protein-protein interaction screens in the yeast Saccharomyces cerevisiae [3,4,8-11]. Sharan *et al*. [12,13] developed a probabilistic model for protein complexes in a single species and a model for the conservation of complexes between two species. Based on the assumption that proteins in the same pathway are typically present or absent in a genome as a group, Pellegrini *et al*. [14] detected the conserved complexes across two species. Methods based on integrated multiple information (e.g. functional annotations for proteins, protein structures, gene expression, *et al*.) have been proposed [5,15]. Dezso *et al*. [5] believe that the cellular role and the essentiality of a protein complex may largely be determined by a small group of protein subunits that display a high mRNA coexpression pattern, belong to the same functional class, and share the same deletion phenotype and cellular localization. However, the relation between protein interactions and gene coexpressions may be very complicated with a high rate of false positive in the protein interaction data generated by high-throughput methods.

At present, a general trend is to represent the protein-protein interactions as a graph and to apply suitable graph algorithms to extract necessary information [16]. There have been several graph clustering approaches proposed to detect protein complexes, including SPC (Super paramagnetic clustering) [17], RNSC (Restricted Neighborhood Search Clustering) [18], MCODE (Molecular Complex Detection) [19,20], DPClus [16], LCMA (Local Clique Merging Algorithm) [21], CFinder [22], and STM (Signal Transduction System) [23]. For later comparisons of our proposed algorithm with these algorithms, we give a brief description and discussion on each of these algorithms.

SPC [17] is a hierarchical clustering algorithm that simulates a ferromagnetic model with physical properties subject to fluctuation at nonzero temperature. Algorithm SPC identifies vertices belonging to a highly connected subgraph. However, as a disadvantage, SPC is sensitive to noisy data [24]. In fact, to our knowledge, all methods of predicting protein-protein interactions cannot avoid yielding a non-negligible amount of noise (false positives).

RNSC [18] is a cost-based clustering algorithm, which partitions the vertices of a graph into clusters based on a cost function that is assigned to each partitioning. It starts from an initial random solution and iteratively moves a vertex from one cluster to another to decrease the total cost of clusters. It ends up when some moves have been reached without decreasing the cost function. RNSC is a randomized algorithm and its results depend heavily on the quality of the initial clustering.

MCODE [19,20] is a density-based local search algorithm that operates in three stages: vertex weighting, complex prediction, and optionally post-processing. First, it assigns a weight to each vertex based on its local neighborhood density. Then, it seeds a complex with the highest weighted vertex and recursively moves outward from the seed vertex. A new vertex is added to the complex if its weight is larger than a given threshold. Then, it filters or adds proteins in the clusters by certain connectivity criteria. However, MCODE cannot guarantee that the predicted clusters are highly connected to each other, since the highly weighted vertices may not be highly connected to each other.

Most importantly, SPC, RNSC and MCODE cannot generate overlapping protein complexes, and require that each vertex belong to one specific cluster. In practice, a protein may be involved in multiple complexes and have more than one biological function. For example, in the CYGD database [25], the ratio of the number of proteins in known protein complexes over the sum of the sizes of these complexes is 2750/8932. Therefore, it is practically important to develop algorithms that identify overlapping protein complexes. The DPClus [16] clustering algorithm is based on density and periphery tracking and can detect both non-overlapping clusters and overlapping clusters. To generate overlapping clusters, DPClus extends the non-overlapping clusters by adding their neighbors in the original graph (rather than in the remaining graph). It starts at a highest weighted vertex and grows gradually by adding vertices from its neighbors. It uses two parameters, density *d*_*k *_and cluster property *cp*_*nk*_. A vertex added to a cluster must satisfy two conditions: 1) its addition does not cause the density *d*_*k *_of the cluster to fall below a given threshold *d*_*in*_; and 2) its *cp*_*nk *_is larger than another given threshold *cp*_*in*_.

LCMA [21] generates overlapping clusters based on local clique merging. It first locates local cliques for each vertex of the graph then merges the detected local cliques according to their affinity to form maximal dense subgraphs.

CFinder [22] is a tool of detecting overlapping clusters based on the Clique Percolation Method (CPM) [26]. CPM defines a protein complex as a union of all *k*-cliques that can be reached from each other through a series of adjacent *k*-cliques (two *k*-cliques are adjacent if they share exactly *k *- 1 vertices). Results of CFinder are highly correlated to the value of the parameter *k*. Larger values of *k *correspond to smaller subgraphs of higher density.

STM [23] models protein interaction networks as dynamic signal transduction systems, and demonstrates the signal transduction behavior of perturbations by proteins statistically. STM allows overlapping of output clusters and identifies clusters of large size, arbitrary shape, and low density. However, unexpected huge clusters may also be generated in its post-process of merging.

In this paper, we propose a clustering algorithm, which follows the general framework of the algorithm DPClus [16] but is based on a new topological structure of complexes. By a careful study of the structures of known complexes, we discover that most complexes have a very small diameter and a very small average vertex distance. Also observing that vertex distance along would not precisely determine the desired complex structures, we propose a new topological structure of complexes that is the combination of vertex distance and subgraph density. Following the general approach of expanding clusters started with seeded vertices, as what DPClus did, we develop an algorithm IPCA for detecting protein complexes based on the new topological structure. We apply the algorithm IPCA to the protein interaction network of yeast, and identify many well-known protein complexes. We compare IPCA with the six competing previous methods DPClus, CFinder, LCMA, MCODE, RNSC and STM. The clusters generated by each method are compared to the known protein complexes. The results of the comparisons show that much more experimentally determined complexes are recalled by IPCA than by other six methods. In addition, IPCA is robust against the high rate of false positives and false negatives in the protein interaction networks. Thus, the algorithm IPCA can be used to identify new protein complexes in protein interaction networks of various species and provide references for biologists in their research on protein complexes.

Before we present our algorithm, we would like to discuss the difference between our algorithm IPCA and the previously proposed algorithm DPClus [16]. The algorithm IPCA follows the general approach of cluster expanding based on seeded vertices, as what DPClus did. However, the rules of IPCA for expanding clusters and weighting vertices are somewhat different from that of DPclus especially they target a different topological structure for the resulted clusters. In particular, the algorithm DPClus identifies subgraphs that satisfy a density condition (i.e., *d*_*k*_) and certain cluster connectivity property (i.e., *cp*_*nk*_), while the algorithm IPCA looks for subgraph structures that have a small diameter (or a small average vertex distance) and satisfy a different cluster connectivity-density property (i.e., *IN*_*vK*_). Also, the algorithm IPCA computes the vertex weights only once, *based on the original input graph*. On the other hand, once a new cluster is identified, the algorithm DPClus removes the cluster and re-computes the vertex weights *based on the remaining subgraph*. We believe that our approach is biologically more meaningful: the selection of a seeded vertex for a cluster is based on vertex weights, which should be measured by the original protein network because the cluster is a dense structure in the original network. On the other hand, a remaining subgraph in the process of DPClus may have lost some useful biological information because the algorithm re-computes the vertex weights based on the remaining subgraph. A byproduct of our approach is that our algorithm is more efficient because it avoids the recomputation.

### The proposed algorithm

A protein interaction network is represented as an undirected simple graph *G*(*V*, *E*) with proteins as vertices and protein interactions as edges. Previous works [16-21,27] have revealed that protein complexes in a protein interaction network generally correspond to dense regions (dense subgraphs, or simply *clusters*). Most density-based clustering algorithms, such as DPClus [16], first generate a seed vertex and extend from the seed vertex by adding new vertices. The performance of such algorithms depends heavily on the quality of the seeds and the criterion of extending, especially the latter.

In this section, we propose a new extending model by analyzing the topology of the complexes in the protein interaction network of Saccharomyces cerevisiae. The protein interaction network of Saccharomyces cerevisiae is downloaded from MIPS (Munich Information Center for Protein Sequences) database [28]. We remove all the self-connecting interactions and repeated interactions. The final network includes 4546 yeast proteins and 12319 interactions. The average clustering coefficient of the final network is 0.4, the network diameter is 13, and the average vertex distance is 4.42. We also collect from the MIPS database protein complexes annotated for Sacchromyces cerevisiae [28]. There are 216 manually annotated complexes that consist of two or more proteins. The largest complex contains 81 proteins, the smallest complex contains 2 proteins, and the average size of all the complexes is 6.31. For each protein complex, we analyze its topology in the network of Sacchromyces cerevisiae. Of the 216 protein complexes, 118 are connected (a protein complex is connected if there is a path connecting every pair of vertices in the complex). For a connected protein complex, its diameter is defined to be the maximum shortest path length between any pair of vertices in it. A non-connected protein complex can be divided into connected components. Most of the non-connected protein complexes can be divided into a large component and one or two separated proteins. For a non-connected protein complex, we define its diameter to be the maximum diameter over all its connected components. We calculate the diameter and average shortest path length for each of the 118 connected protein complexes and for each of the 98 non-connected protein complexes.

As shown in Table 1, 94.91% of the connected complexes have their diameter bounded by 2, and 99.15% of the connected complexes have their average shortest path length bounded by 2. There is only one connected complex in which the average shortest path length is larger than 2, which is 2.047. The average shortest path length of all the non-connected complexes is bounded by 2.5, the longest one is 2.409. 93.88% of the non-connected complexes have their average shortest path length bounded by 2, and 82.66% of the non-connected complexes have their diameter bounded by 2. This fact matches the observation that the protein interaction networks have the small-world property [29,30]. The analysis on the statistical data shows that the length of the shortest path between each pair of vertices in most of the complexes is bounded by 2. With this important observation, we believe that the length of the diameter and the average length of shortest paths are important topological parameters for detecting protein complexes.

**Table 1:** Diameter and average length of the shortest paths of protein complexes

Complexes(216)	Diameter of the Complex			Average Length of the shortest paths		
	
	Diameter	Number	Proportion	Length	Number	Proportion
Connected(118)	*D *≤ 2	112	94.91%	*L *≤ 2	117	99.15%
	D = 3	5	4.24%	L = 2.047	1	0.85%
	D = 4	1	0.85%			

Non-connected(98)	*D *≤ 2	81	82.66%	*L *≤ 2	92	93.88%
	D = 3	7	7.14%	2 <*L *≤ 2.5	6	6.12%
	D = 4	7	7.14%			
	D = 5	3	3.06%			

In the following discussion, we denote by *SP*(*K*) the diameter of a graph *K *(i.e., the largest length of a shortest path between a pair of vertices in *K*), and by *ASP*(*K*) the average length of all the shortest paths between each pair of vertices in *K*. Since the discussions for *SP*(*K*) and *ASP*(*K*) are similar, our discussion will be mainly focused on *SP*(*K*).

**Figure 1:**
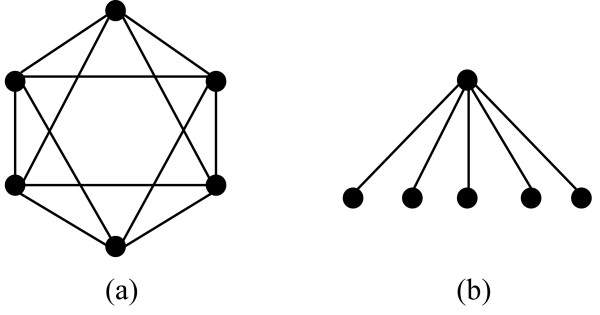
**Two examples of graphs with *SP *= 2**. Graphs with the same diameter can have very different topologies. To distinguish different topologies of graphs with the same diameter, we can use the parameter *IN*_*vK*_. For example, the two graphs in this figure both have diameter 2. However, for all vertices *v *in the first graph, the value *IN*_*vK' *_is 4/5; while for five of the six vertices in the second graph, the value *IN*_*vK' *_is 1/5 (where we define *K' *= *K *- *v*).

As shown in Figure 1, graphs with the same diameter can have very different topologies. To distinguish different topologies of graphs with the same diameter, we need another control parameter. For a dense graph, a vertex is connected to most of the vertices in the graph. On the other hand, in a sparse graph a vertex may be connected to only a few vertices in the graph. We introduce a new concept to measure how strongly a vertex *v *is connected to a subgraph *K*: the *interaction probability IN*_*vK *_of a vertex *v *to a subgraph *K*, where *v *∉ *K*, is defined by

(1) $$IN_{vK}=\frac{m_{vK}}{n_{K}}$$

where *m*_*vK *_is the number of edges between the vertex *v *and *K*, and *n*_*K *_is the number of vertices in *K*. We discuss the relationship between the parameter *IN*_*vK *_and the two parameters *d*_*K *_and *cp*_*vK *_introduced in the algorithm DPClus [16]. According to [16], the *density d*_*K *_of a subgraph *K *is defined as *d*_*K *_= 2*m*_*K*_/(*n*_*K*_(*n*_*K *_- 1)), where *m*_*K *_is the number of edges in the subgraph *K*, and the *cluster property cp*_*vK *_of a vertex *v *with respect to the subgraph *K *is defined as *cp*_*vK *_= *m*_*vK*_/(*d*_*K*_*n*_*K*_). By the expressions, our parameter *IN*_*vK *_is similar to the parameter *cp*_*vK *_(differing by a factor of *d*_*K*_). Moreover, the following theorem shows that the parameter *IN*_*vK *_is also closely related to the density *d*_*K*_.

**Theorem**. Let *K *be a subgraph of a graph *G*. If for every vertex *v *in *K*, we have *IN*_*vK' *_≥ *t*_0_, where *K' *= *K *- *v *and *t*_0 _is a fixed constant, then *d*_*K *_≥ *t*_0_.

PROOF. By the conditions given in the theorem, for all vertices *v *in *K*, we have (where *K' *= *K *- *v*, *m*_*vK' *_is the number of edges between *v *and *K'*, and *n*_*K' *_is the number of vertices in the subgraph *K'*),

*IN*_*vK' *_= *m*_*vK'*_/*n*_*K' *_≥ *t*_0_

Therefore, *m*_*vK' *_≥ *n*_*K'*_*t*_0_. Add this over all vertices *v *in *K*, and note that ∑_*v*∈*K*_*m*_*vK' *_= 2*m*_*K*_, where *m*_*K *_is the total number of edges in *K*, and that *n*_*K' *_= *n*_*K *_- 1, we have

$$2m_{K}=\sum_{v\in K}m_{vK^{\prime }}\geq \sum_{v\in K}n_{K^{\prime }}t_{0}=n_{K}(n_{K}-1)t_{0}$$

This gives *d*_*K *_= 2*m*_*K*_/(*n*_*K*_(*n*_*K *_- 1)) ≥ *t*_0_, and proves the theorem.   □

By the above theorem, a lower bound on the parameter *IN*_*vK' *_for every vertex *v *in a subgraph *K *will also provide a lower bound on the density *d*_*K *_of the subgraph *K*. Next we show that the parameter *IN*_*vK *_can help distinguishing subgraph structures that are indistinguishable by their diameters. Consider the two graphs in Figure 1 again. Although both graphs have diameter 2, for all vertices *v *in the first graph, the value *IN*_*vK' *_is 4 = 5; while for five of the six vertices in the second graph, the value *IN*_*vK' *_is 1/5 (where we define *K' *= *K *- *v*).

Our algorithm IPCA looks for complex structures whose topological structure is controlled by the two parameters *SP*(*K*) and *IN*_*vK*_. More specifically, we look for complex structures whose diameter is controlled by the parameter *SP*(*K*) and whose density and cluster property are controlled by the parameter *IN*_*vK*_.

*Definition 1*. Let *T*_*in *_be a threshold ranging between 0 and 1, let *d *be a positive integer, and let *K *be a subgraph. A vertex *v *∉ *K *is a (*K*, *T*_*in*_, *d*)*-vertex *if the following two conditions are satisfied (where *K *+ *v *denotes the subgraph induced by *K *and *v*):

1. *IN*_*vK *_≥ *T*_*in*_; and

2. *The *(*SP *≤ *d*)*-Version*: *SP*(*K *+ *v*) ≤ *d *(or *The *(*ASP *≤ *d*)*-Version*: *ASP*(*K *+ *v*) ≤ *d*)

Note that there are actually two versions for the definition of a (*K*, *T*_*in*_, *d*)-vertex in terms of condition 2: one uses the condition *SP*(*K *+ *v*) ≤ *d *(i.e., the (*SP *≤ *d*)-Version), and the other uses the condition *ASP*(*K *+ *v*) ≤ *d *(i.e., the (*ASP *≤ *d*)-Version).

**Figure 2:**
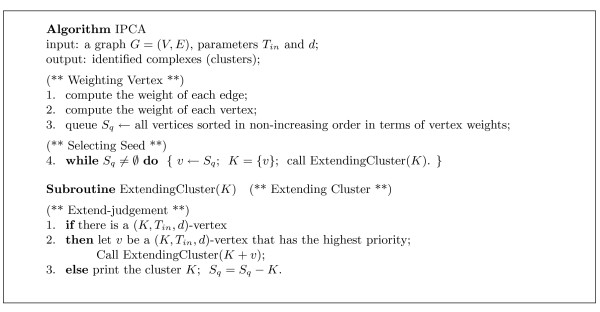
**The description of IPCA algorithm**. IPCA algorithm extends clusters based on (*K*, *T*_*in*_, *d*)-vertices. If the algorithm uses the (*SP *≤ *d*)-Version in the conditions of Definition 1, we will say that "the algorithm uses *SP *≤ *d*". Similarly, if the algorithm uses the (*ASP *≤ *d*)-Version in the conditions of Definition 1, we will say that "the algorithm uses *ASP *≤ *d*".

Our clustering algorithm IPCA that extends clusters based on (*K*, *T*_*in*_, *d*)-vertices is given in Figure 2. If the algorithm uses the (*SP *≤ *d*)-Version in the conditions in the definition, we will say that "the algorithm uses *SP *≤ *d*". Similarly, if the algorithm uses the (*ASP *≤ *d*)-Version in the conditions in the definition, we will say that "the algorithm uses *ASP *≤ *d*".

The algorithm IPCA can be divided into four major parts: *Weighting Vertex*, *Selecting Seed*, *Extending Cluster*, and *Extend-judgment*. The input to the algorithm is an undirected simple graph.

#### Weighting Vertex

Our procedure of vertex weighting is the same as that of the DPClus algorithm [16]. For an input graph *G *= (*V*, *E*), we assign the weight of an edge [*u*, *v*] to be the number of neighbors shared by the vertices *u *and *v*. We define the weight of each vertex to be the sum of the weights of its incident edges. After all vertices are assigned weights, however (this is different from DPClus), we also sort in non-increasing order the vertices by their weights and store them in a queue *S*_*q *_(vertices of the same weight are ordered in terms of their degrees). The complexity of calculating edge weights and vertex weights is *O*(|*V*||*E*|), and the complexity of sorting all vertices by their weights is *O*(|*V*| log |*V*|).

#### Selecting Seed

The notion that vertex weight is a good measure for selecting seeds has been adopted by DPClus [16] and MCODE [19]. Here, we also pick the highest weighted vertices as the seeds. Our procedure proceeds as follows. We pick the first vertex in the queue *S*_*q *_and use it as a seed to grow a new cluster. Once the cluster is completed, all vertices in the cluster are removed from the queue *S*_*q *_and we pick the first vertex remaining in the queue *S*_*q *_as the seed for the next cluster. There are a number of important differences between this seed selection procedure and the one used in the DPClus algorithm [16]. First, our procedure computes the vertex weight for each vertex only once, based on the original graph; while the DPClus algorithm recomputes the vertex weight for each remaining vertex after a cluster is removed, *based on the remaining subgraph*. We feel that our approach is biologically more meaningful because a complex is a dense structure in the original protein network thus its seed vertex should be measured in terms of the original graph. On the other hand, a remaining subgraph *G' *during the process of DPClus may have lost certain biological information (e.g., interactions between the vertices in *G' *and vertices not in *G' *have been removed). Computationally, our approach also has the advantage of being more efficient. Moreover, our approach has also helped for generating overlapping complexes. This is because the vertices of the generated clusters are only removed from the queue *S*_*q*_, but not from the original graph *G*.

#### Extending Cluster

A cluster *K *is extended by adding vertices recursively from its neighbors according to the *priority*. The priority of a neighbor *v *of *K *is determined by the value *IN*_*vK*_. This procedure is similar to the one proposed in DPClus [16], except that we do not use "fine-tuning" to sort the neighbors. Whether a high priority vertex *v *is added to the cluster is determined by the *Extend-judgment *test that tests if *v *is a (*K*, *T*_*in*_, *d*)-vertex. Only when the candidate vertex *v *is a (*K*, *T*_*in*_, *d*)-vertex, can it be added to the cluster. Once the new vertex *v *is added to the cluster, the cluster is updated, i.e., the neighbors of the new cluster are re-constructed and the priorities of the neighbors of the new cluster are re-calculated, and the algorithm goes recursively with the new cluster. The complexity of generating a candidate vertex from the neighbors of the cluster *K *is *O*(*n*_*K*_*n*_*N*(*K*) _+ *n*_*N*(*K*) _log *n*_*N*(*K*)_), where *n*_*N*(*K*) _is the number of neighbors of *K*.

#### Extend-judgment

Whether a candidate vertex *v *is added to a cluster *K *is determined by the two conditions given in Definition 1. First, we calculate the value *IN*_*vK*_. The vertex will not be added to the cluster if the value *IN*_*vK *_is less than *T*_*in*_. If the vertex *v *passes this test, then depending on whether using *SP *≤ *d *or *ASP *≤ *d*, the algorithm computes the diameter of the graph *K *+ *v *or the average length of the shortest paths between pairs of vertices in *K *+ *v*, and compares the value with the parameter *d*. If the computed value is bounded by *d*, then the vertex *v *is added to the cluster. If the vertex *v *fails any of these tests, then the next highest priority neighbor of the cluster is tested, and so on. If all neighbors fail the tests, then the cluster cannot be further extended, and a complete cluster is formed whose vertices are removed from the queue *S*_*q*_. In this paper, *d *= 2 is used according to our previous analysis. The complexity of testing whether a candidate vertex is added to a cluster is *O*($n_{K}^{2}$).

We remark that our algorithm IPCA guarantees that no two generated clusters would be the same: a seed vertex *v *for a new cluster is selected such that *v *does not belong to any of the previously constructed clusters. In fact, any two clusters constructed by the algorithm IPCA should be expected to be sufficiently different. To see this, let *C*_2 _be a cluster seeded at *v*_2 _that is constructed after a cluster *C*_1_. If the two clusters *C*_1 _and *C*_2 _are largely overlapping, then intuitively, the vertex *v*_2 _is closely and densely connected to many vertices in *C*_1_. Thus, during the construction of the cluster *C*_1_, the vertex *v*_2 _would have a large chance to be included in *C*_1 _and would have not become a seed for the later cluster *C*_2_.

The time complexity of the entire algorithm IPCA depends on the number and the size of predicted clusters. The running time of IPCA is given in the next section.

## Results and Discussion

The protein interaction network of Sacchromyces cerevisiae is downloaded from the MIPS database [31]. After removal of all the self-interactions and repeated interactions, the final network includes 4546 proteins and 12319 interactions. We apply the proposed algorithm IPCA to this network. In the following subsections, we discuss the effect of the value *T*_*in *_on clustering, compare the predicted clusters with the known complexes, evaluate the significance of the predicted clusters, and analyze the robustness and efficiency of the algorithm IPCA. We will also compare the algorithm IPCA to six competing previous methods for their performance of identifying protein complexes. The comparisons are also performed on protein interaction networks and random networks.

### The effect of *T*_*in *_on clustering

**Figure 3:**
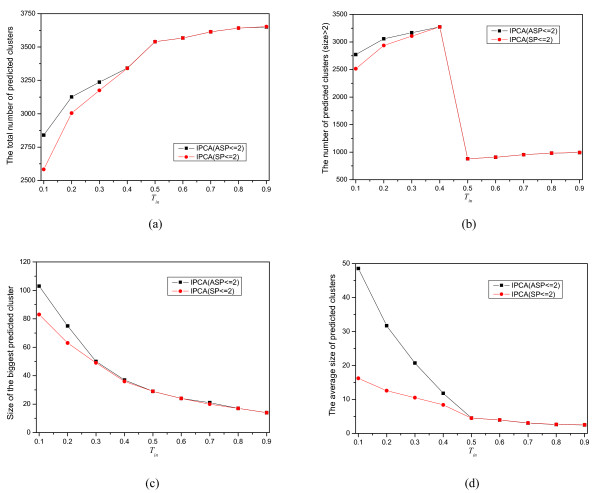
**The effect of *T*_*in *_on clustering**. Nine sets are generated from the yeast network by IPCA using *SP *≤ 2 and *T*_*in *_= 0.1, 0.2,...,0.9, and nine sets are generated by IPCA using *ASP *≤ 2 and *T*_*in *_= 0.1, 0.2,...,0.9. (a) the total number of predicted clusters, (b) the number of the predicted clusters with *size *> 2, (c)size of the biggest predicted cluster, (d) The average size of the predicted clusters.

To understand how the value of *T*_*in *_influences the outcome of the clustering, we generate 18 sets of clusters by using *SP *≤ 2 and *ASP *≤ 2 with *T*_*in *_= 0.1, 0.2,..., 0.9 from the protein interaction network of yeast. The effect on the predicted clusters with different *T*_*in *_is given in Figure 3. Figure 3(a) shows that the total number of the predicted clusters is increasing as *T*_*in *_increases. However, in Figure 3(b), there is a abrupt decrease at *T*_*in *_= 0.5. This is probably caused by the Hub structures in the protein interaction network. When *T*_*in *_= 0.5, these Hub structures are decomposed into complexes that consist of only 2 proteins.

Figure 3(c) shows that the size of the biggest cluster is decreasing as *T*_*in *_increases. The same trend of the average size of the predicted clusters is shown in Figure 3(d). With the increasing of *T*_*in*_, the probability of neighbors added to the cluster is decreasing. Thus, the size of the predicted clusters is also decreasing. From Figure 3, we can see that there is almost no difference for the clusters generated by using *SP *≤ 2 or by using *ASP *≤ 2 when *T*_*in *_≥ 0.5. More and larger clusters are generated by using *ASP *≤ 2 than by using *SP *≤ 2 with the same *T*_*in *_when it is smaller than 0.5.

### Comparison with the known complexes

**Figure 4:**
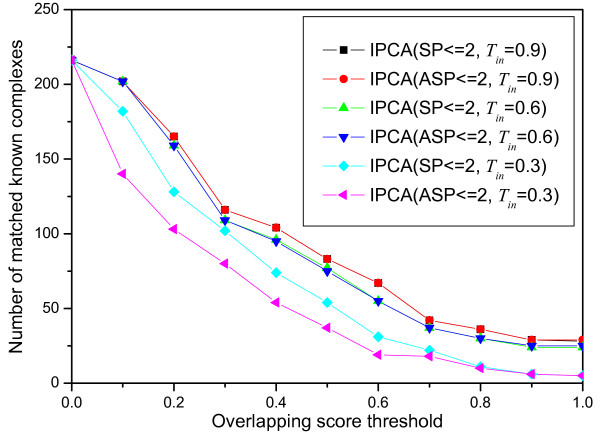
**Comparison of the predicted clusters with the known complexes**. The number of matched known complexes with respect to different overlapping scores for different sets generated by IPCA using different parameters.

To evaluate the effectiveness of the algorithm IPCA for detecting protein complexes, we compare the predicted clusters produced by the algorithm with known protein complexes in MIPS yeast complex database [32]. There are 216 manually annotated complexes considered as the gold standard data that each consists of two or more proteins. Here, we use the same scoring scheme used in [16,19] to determine how effectively a predicted cluster (*Pc*) matches a known complex (*Kc*). The *overlapping score OS*(*Pc*, *Kc*) between a predicted cluster *Pc *and a known complex *Kc *is calculated by the following formula:

(2) $$OS(Pc,Kc)=\frac{{\left|V_{Pc}\cap V_{Kc}\right|}^{2}}{\left|V_{Pc}\right|\cdot \left|V_{Kc}\right|}$$

where |*V*_*Pc *_∩ *V*_*Kc*_| is the size of the intersection set of the predicted cluster and the known complex, |*V*_*Pc*_| is the size of the predicted cluster and |*V*_*Kc*_| is the size of the known complex. A known complex *Kc *that has no proteins in a predicted cluster *Pc *has *OS*(*Pc*, *Kc*) = 0 and a known complex *Kc *that perfectly matches a predicted cluster *Pc *has *OS*(*Pc*, *Kc*) = 1. A known complex and a predicted cluster are considered as a match if their overlapping score is equal to or larger than a specific threshold. The numbers of matched known complexes with respect to different overlapping score threshold (from 0 to 1 with a 0.1 increment) are shown in Figure 4. The best matching result is obtained when *T*_*in *_= 0.9 for both *SP *≤ 2 and *ASP *≤ 2. There are 165 known complexes matched when the overlapping score threshold is 0.2. There are 28 known complexes matched perfectly. When *T*_*in *_≥ 0.5, the number of matched known complexes is almost the same for *SP *≤ 2 and *ASP *≤ 2. When *T*_*in *_≤ 0.5, the number of matched known complexes is larger for *SP *≤ 2 than for *ASP *≤ 2. The probability that a known complex is matched perfectly by a cluster in which proteins are picked up randomly is determined by the size of the network and the known complex. The probability that a known complex with size = 3 matches perfectly by a cluster selected randomly in the yeast network used in this paper is 6.39 * 10^-11^. It is very obvious that more known complexes matched by the predicted clusters implies that the algorithm is more effective to detect complexes. Sensitivity and specificity are two important aspects to estimate the performance of algorithms for detecting protein complexes. *Sensitivity *is the fraction of the true-positive predictions out of all the true predictions, defined by the following formula:

(3) $$Sn=\frac{TP}{TP+FN}$$

where *TP *(true positive) is the number of the predicted clusters matched by the known complexes with *OS*(*Pc*, *Kc*) ≥ 0.2, and *FN *(false negative) is the number of the known complexes that are not matched by the predicted clusters. *Specificity *is the fraction of the true-positive predictions out of all the positive predictions, defined by the following formula:

(4) $$Sp=\frac{TP}{TP+FP}$$

where *FP *(false positive) equals the total number of the predicted clusters minus *TP*. According to the assumption in [19], a predicted cluster and a known complex are considered to be matched if *OS*(*Pc*, *Kc*) ≥ 0.2. Here, we also use 0.2 as the matched overlapping threshold.

Another integrated method, called the *f-measure*, has been used in [21,20], which is defined as follows:

(5) $$f\text{-measure}=\frac{2*Sn*Sp}{Sn+Sp}$$

As pointed out in [21,20], the *f*-measure of each method can only be taken as a comparative measure rather than its real values, because the reference set MIPS is incomplete and some predicted clusters that may be true complexes could be regarded as false positives (*FP*) if they do not match with the known complexes. Nevertheless, it is still reasonable to consider a method more effective if it detects more known complexes. The coverage of the known complexes is defined by the following formula:

(6) $$Cov(Kc)=\frac{N_{Kc}-FN}{N_{Kc}}$$

where *N*_*Kc *_is the total number of known complexes. The sensitivity, the specificity, the *f *-measure, and the coverage of the clusters generated by the algorithm IPCA using different parameters are shown in Table 2. The sensitivity of the clusters generated by IPCA is about 0.8 and very close to 0.9 when *T*_*in *_> 0.5. The value *TP *is 4 times more than the value *FN*, which implies that the clusters generated by IPCA are reliable. The specificity of an algorithm represents the real positive proportion of all the predicted clusters. As shown in Table 2, the specificity of the algorithm IPCA is larger than 0.1, but smaller than 0.2. The low specificity is probably because of the incompleteness of the known complexes. The *f*-measure takes into account of both the sensitivity and the specificity, and is determined by the larger one. In this experiment, the *f*-measure is mostly influenced by the sensitivity. The sensitivity is about 1.6 ~ 1.8 times of the specificity. The coverage of the clusters generated by IPCA increases with the increasing of *T*_*in*_. Especially, an obvious increase appears when *T*_*in *_≥ 0.5. In Table 2, we can observe that the sensitivity, the specificity, the *f*-measure, and the coverage of the clusters generated by IPCA using *SP *≤ 2 are slightly larger than those generated by IPCA using *ASP *≤ 2.

**Table 2:** The Sensitivity(*S*_*n*_), Specificity(*S*_*p*_), *f*-measure(*f*) and Coverage(*Cov*)of the predicted clusters generated by IPCA using different parameters

Parameter	*SP *= 2				*ASP *= 2			
	
	*S*_*n*_	*S*_*p*_	*f*	*Cov*	*S*_*n*_	*S*_*p*_	*f*	*Cov*
*T*_*in *_= 0.1	0.764	0.123	0.211	0.546	0.715	0.122	0.208	0.361
*T*_*in *_= 0.2	0.822	0.140	0.239	0.579	0.763	0.134	0.228	0.398
*T*_*in *_= 0.3	0.862	0.173	0.288	0.593	0.788	0.129	0.222	0.477
*T*_*in *_= 0.4	0.883	0.184	0.304	0.625	0.804	0.110	0.194	0.583
*T*_*in *_= 0.5	0.864	0.106	0.189	0.727	0.864	0.106	0.189	0.727
*T*_*in *_= 0.6	0.900	0.144	0.248	0.736	0.901	0.145	0.250	0.736
*T*_*in *_= 0.7	0.897	0.125	0.219	0.759	0.897	0.125	0.219	0.759
*T*_*in *_= 0.8	0.895	0.119	0.210	0.764	0.895	0.119	0.210	0.764
*T*_*in *_= 0.9	0.895	0.119	0.209	0.764	0.894	0.118	0.209	0.764

### Comparison of protein interaction networks and random networks

**Figure 5:**
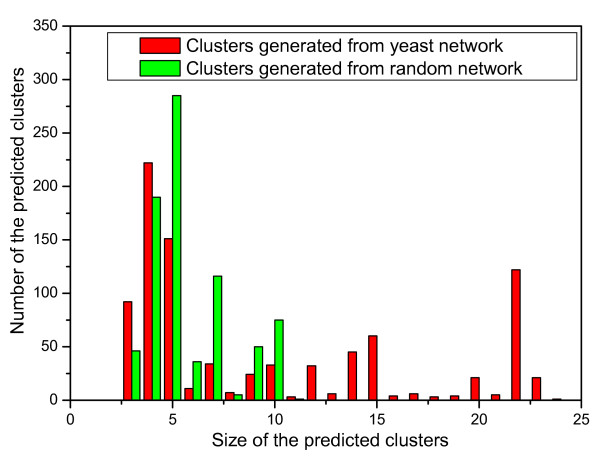
**The size distribution of the predicted clusters(*size *> 2)**. The distribution of the predicted clusters (*size *> 2) generated by IPCA using *T*_*in *_= 0.6 from the yeast network and the random graph with respect to size.

**Figure 6:**
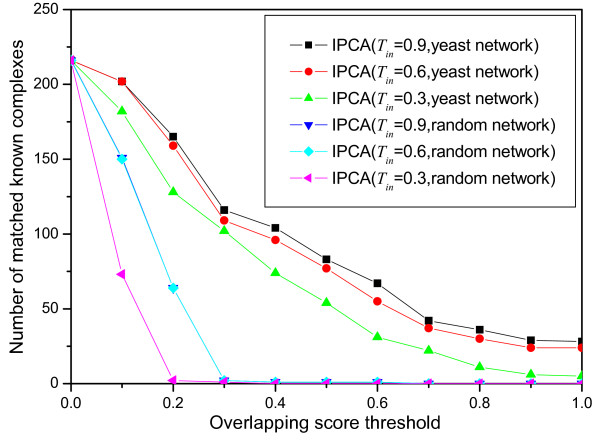
**The number of known complexes matched by the clusters from the yeast network and the random network**. The number of matched known complexes with respect to different overlapping scores for different sets generated by IPCA using different parameters from the yeast network and the random network.

To evaluate whether the clusters generated by the algorithm IPCA from the protein interaction network are biologically significant, we experiment the algorithm on the protein interaction network of yeast and on a random network of a similar structure. The random network, which has the same size and the same degree distribution as the yeast network, is obtained by shu^2^ing the edges between vertices in the yeast network. More clusters are generated from the random network than from the yeast network, and the clusters generated from the random network have less proteins than those generated from the yeast network. Figure 5 shows the size distributions of the clusters generated by IPCA using *T*_*in *_= 0.6 from the yeast network and from the random network. As shown in the Figure, the predicted clusters identified in the yeast network are in various sizes from 2 to 25, while those in the random network are in various size from 2 to 10. Many small clusters are detected in the random network. To evaluate whether all these small clusters in the random network are significant, we compare them with the known complexes. As shown in Figure 6, while there are more than 100 known complexes matched by the predicted clusters identified in the yeast network when the overlapping score threshold is larger than 0.2, there are almost no known complexes matched by the predicted clusters identified in the random network when the overlapping score threshold is larger than 0.2. This result shows that the random network destroys the biological intrinsic character in the protein interaction network, though it has the same degree distribution as the original yeast network.

### Comparison of IPCA and other methods

Since there have been protein complexes that were experimentally determined, a good protein complexes detecting algorithm should identify these known complexes as many as possible. Table 3 shows the numbers of known complexes matched to the clusters generated by IPCA and by other six previous known methods: DPClus [16], CFinder [22], LCMA [21], MCODE [19], RNSC [18], and STM [23].

**Table 3:** Comparison of the number of known complexes matched by the predicted clusters generated by IPCA and other previous algorithms

Overlapping Score Threshold	Algorithms						
	IPCA	DPClus(Ov)	CFinder	LCMA	MCODE	RNSC	STM
	*T*_*in *_= 0.9	*CP*_*in *_= 0.5	*k *= 3	*NA *= 0.4	*V WP *= 0.1	*K *= 1200	No Merging
	*SP *≤ 2	*D*_*in *_= 0.9			*Fluff *= 0		
*OS *≥ 0.1	202	191	77	136	60	170	116
*OS *≥ 0.2	165	128	54	105	35	115	57
*OS *≥ 0.3	116	85	42	89	27	92	26
*OS *≥ 0.4	104	74	33	74	24	71	18
*OS *≥ 0.5	83	52	25	55	17	52	13
*OS *≥ 0.6	67	41	19	45	13	26	10
*OS *≥ 0.7	42	25	13	26	11	17	6
*OS *≥ 0.8	36	20	7	20	8	11	5
*OS *≥ 0.9	29	15	5	13	4	6	4
*OS *= 1.0	28	15	5	12	4	6	4

Though the method DPClus can generate clusters with two modes (non-overlapping and overlapping), it does not include the overlapping option at present. We generated by ourselves ten sets with non-overlapping mode using DPClus. Another ten sets with overlapping mode were provided by an author (Md Altaf-UI-Amin) of DPClus. When *CP*_*in *_= 0.5 and *D*_*in *_= 0.9, DPClus gets the best matching results. Since it is more significant to detect overlapping protein complexes, we consider the best matching results generated by DPClus with overlapping mode.

The method CFinder gets the best matching results by setting *k *= 3.

The method LCMA gets the best matching results by setting *NA *= 0.4.

For the method MCODE, there are 840 parameter combinations, and most of them have similar matching results. The method MCODE gets the best matching results when the "haircut" option is not used and when *VWP *= 0.1 and *Fluff *= 0.

The method RNSC gets the best matching results when the number of the predicted clusters is about 1200. The authors of the method STM have shown that the method gets the best performance when the merge threshold value is 1.0. However, a huge cluster that includes 4358 proteins and 85 clusters whose sizes vary from 2 to 7 are generated when the merge threshold value is 1.0. Of all the 86 clusters, only 4 clusters match known complexes with the overlapping sore *OS *≥ 0.2. Thus, we consider the matching results of the clusters not merged for the method STM.

As shown in Table 3, the clusters generated by IPCA match more known complexes than all six other methods for all different overlapping score thresholds. When *OS *> 0.2, the number of matched known complexes by IPCA is about 1.2 times more than that by DPClus, LCMA and RNSC. With the same overlapping score threshold, the number of matched known complexes by IPCA is about 2.7 times more than that by CFinder, and about 4.2 times more than that by MCODE and STM. When *OS *= 1, there are no more than 10 matched known complexes by CFinder, MCODE, RNSC, and STM. On the other hand, there are 28 known complexes matched perfectly by the clusters of IPCA.

The results show that IPCA outperforms all these six previous methods on the performance of identifying protein complexes.

As an additional and interesting example, we compare the performance of IPCA and DPClus for generating large complexes. Since small complexes (e.g., complexes consisting of no more than 2 proteins) have been represented in the protein-protein interaction data, one may be more interested in identifying large complexes. For this, we compare the results generated by IPCA and that generated by DPClus. The comparisons are given in Table 4. As shown in the table, the large clusters (consisting of 3 or more proteins) generated by IPCA match more known complexes than that generated by DPClus for all different overlapping score thresholds. Let *N *be the number of total known complexes that are matched by any generated clusters (consisting of 2 or more proteins) and *N*_≥3 _be the number of known complexes matched by the generated clusters consisting of at least 3 proteins. Then *N*_≥3_/*N *of IPCA is 62.8% and that of DPClus is 58.1%. That is, the clusters generated by IPCA match more known large complexes than that by DPClus.

**Table 4:** Comparison of the number of known complexes matched by the predicted clusters (consisting of 3 or more proteins) generated by IPCA and DPClus.

Overlapping Score(*OS*)	IPCA(*T*_*in *_= 0.9;*SP *≤ 2)	DPClus(Ov;*CP*_*in *_= 0.5;*D*_*in *_= 0.9)
*OS *≥ 0.1	134 (202)	116 (191)
*OS *≥ 0.2	103 (165)	78 (128)
*OS *≥ 0.3	87 (116)	63 (85)
*OS *≥ 0.4	75 (104)	54 (74)
*OS *≥ 0.5	56 (83)	35 (52)
*OS *≥ 0.6	45 (67)	28 (41)
*OS *≥ 0.7	27 (42)	15 (25)
*OS *≥ 0.8	21 (36)	10 (20)
*OS *≥ 0.9	14 (29)	5 (15)
*OS *= 1.0	13 (28)	5 (15)

The number of known complexes matched by the predicted clusters (consisting of 2 or more proteins) is shown in brackets.

### Function Enrichment Analysis

In order to detect the functional characteristics of the predicted clusters, we compare the predicted clusters with known functional classification. The P-value based on hypergeometric distribution is often used to estimate whether a given set of proteins is accumulated by chance. It has been used as a criteria to assign each predicted cluster a main function [18,16]. Here, we also calculate P-value for each predicted cluster and assign a function category to it when the minimum P-value occurrs. The P-value is defied as follows.

(7) $$P=1-\sum_{i=0}^{k-1}\frac{\left(\begin{array}{c} F\\ i \end{array}\right)\left(\begin{array}{c} N-F\\ C-i \end{array}\right)}{\left(\begin{array}{c} N\\ C \end{array}\right)}$$

where *N *is the total number of vertices in the network, *C *is the size of the predicted cluster, *F *is the size of a functional group, and *k *is the number of proteins of the functional group in the predicted cluster. The functional classification of proteins used in this paper was collected from the MIPS Functional Catalog (FunCat) database. FunCat [33] is an annotation scheme of tree-like structure for the functional description of proteins. There are up to 6 levels of increasing specificity and 1360 functional categories in FunCat. We obtained 443 clusters with size ≥ 6 when using *T*_*in *_= 0.6 and obtained 132 clusters with the same size when using *T*_*in *_= 0.9. All these predicted clusters with size ≥ 6 by using *T*_*in *_= 0.6 and *T*_*in *_= 0.9 match well with the known functional categories with P-value < 0.001. As the fact that proteins in the same complex are of similar function, we predicted 7 previously un-characterized proteins in the predicted clusters generated by *T*_*in *_= 0.9 and predicted 50 previously un-characterized proteins in the predicted clusters generated by *T*_*in *_= 0.6. For example, the unknown function protein YOR264w is included in a 7-member cluster, of which six are the cytoskeleton/structural proteins. Thus, we can predict that the function unknown protein YOR264w is also a cytoskeleton/structural protein. All the clusters of size ≥ 6 generated by *T*_*in *_= 0.9 and *T*_*in *_= 0.6 and their main function annotations are given in an additional file 1.

The un-characterized proteins in these clusters are also given in the additional file 1. As the incompleteness of the function annotation, we can also predict new membership for the known complexes and predict new functions for known proteins. As shown in additional file 2, the main function of a 10-member cluster is splicing (11.04.03.01). Seven proteins of the cluster are related to splicing. Other three proteins in the cluster without the function of splicing are all related to mRNA processing (splicing, 5'-, 3'-end processing), which is a higher level of splicing. Thus, we can deduce that the three proteins involved in mRNA processing may be members of the splicing complexes.

### Robustness Analysis

**Figure 7:**
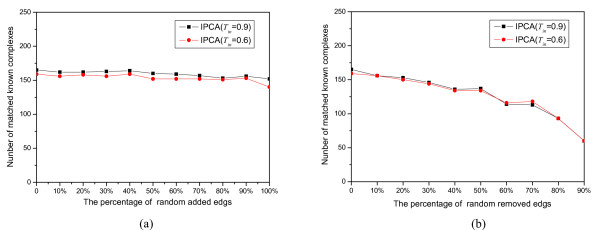
**The robustness of IPCA against random edges addition and removal**. (a) Various proportions of edges added to the protein interaction network randomly, (b) Various proportions of edges removed from the protein interaction network randomly.

In this analysis, we evaluated the robustness of the algorithm IPCA to various levels of graph alterations. Since all the methods of PPIs (Protein-Protein Interactions) prediction are known to yield a non-negligible amount of noise (false positives) and to miss a fraction of existing interactions (false negatives) [24], we tested the robustness of IPCA to false positive by adding edges randomly and to false negatives by removing edges randomly. Proportions of edges (0%, 10%, 20%, 30%,..., 90% and 100%) were added to the yeast protein interaction network randomly, and the same proportions (except that of 100%) of edges were removed from the yeast network randomly. It should be expected that the false positives would not randomly contribute to the formation of dense sub-graphs, and that the number of matched known complexes does not decrease fast with the increasing of false negatives. Figure 7 displays the impact of edge addition and removal on the results of the algorithm IPCA. As one can see, IPCA is barely affected by addition of up to 100% edges. It is also affected faintly by removal of up to 50% edges. It starts to drop perceivably from 60%, and a fast drop starts from 80%. However, there are still 93 known complexes matched to the predicted clusters (*T*_*in *_= 0.9 and *T*_*in *_= 0.6) when 80% edges are removed. The analysis strongly shows that the algorithm IPCA is very robust against the high rate of false positives and false negatives in protein interactions.

### Efficiency Analysis

All experiments in this paper are implemented on a PC with 1.7 GHz processor and 512 M RAM. Table 5 shows the running time of the algorithm IPCA when it generates clusters using different *T*_*in *_values from the yeast network, which consists of 4546 proteins and 12319 interactions. The longest running time is 64 seconds when *T*_*in *_= 0.1, and the shortest running time is 10 seconds when *T*_*in *_= 0.9. The running time is decreasing as *T*_*in *_increases. This is because the probability that proteins added to the clusters is decreasing with the increasing of *T*_*in*_, and because the candidate selection and the judgement whether a candidate can be added to a cluster are time consuming. As a comparison, under the same test environment, the running time of the method DPClus to generate clusters with non-overlapping mode from the same yeast protein interaction network is about 20 minutes.

**Table 5:** Running time of IPCA with various *T*_*in *_(second)

*T*_*in*_	0.1	0.2	0.3	0.4	0.5	0.6	0.7	0.8	0.9
Time(s)	64	53	45	36	21	18	14	12	10

## Conclusion

It is believed that identification of protein complexes is useful to explain certain biological progress and to predict functions of proteins. In this paper, we proposed a new topological structure for protein complexes and developed an algorithm IPCA to identify protein complexes in large protein interaction networks based on the new topological structure. Interaction networks are represented by undirected simple graphs and we generate predicted clusters in the networks by using seed selection and local search. The seeds in the networks are calculated only once, which has reduced the running time of the algorithm effectively. Two parameters, *SP*(*K*) (or *ASP*(*K*)) and *IN*_*vK*_, are used that reflect the statistics of topological structures of the networks. As the accumulation of new complexes and protein-protein interactions, the thresholds of the parameters *SP*(*K*) (or *ASP*(*K*)) and *IN*_*vK *_can be changed easily for generating different types of clusters. Moreover, the algorithm IPCA can generate overlapping protein complexes, which is consistent with the fact that many of the known protein complexes are overlapping. Interesting questions for further research include how many functions a protein can have, how many processes a protein can participate in, and how heavily two protein complexes should overlap with each other.

We applied the algorithm IPCA to the protein interaction network of Sacchromyces cerevisiae. Many well-known complexes were found in the protein interaction network. We predicted the functions for un-characterized proteins and predicted new functions for the known proteins by minimizing the P-values of the predicted clusters. We tested the robustness of our algorithm by adding and removing edges in the network randomly. The results have shown that our algorithm is robust against the high rate of false positives and false negatives in the protein interaction networks. Our algorithm can thus be used to identify new protein complexes in protein interaction networks of various species and to provide references for biologists in their research on protein complexes.

## Methods

The protein interaction data of Sacchromyces cerevisiae was collected from MIPS [31], represented as pairs of interacting proteins. First we removed self-interactions and repeated interactions. The final network includes 4546 yeast proteins and 12319 interactions. We also collected from the MIPS database protein complexes annotated for Sacchromyces cerevisiae [32]. We discarded those consisting of only one protein and the final remaining 216 manually annotated complexes are considered as the gold standard data. The proposed algorithm IPCA has been implemented in C++.

## Authors' contributions

ML developed and implemented the clustering algorithm. JC and JW supervised the work and contributed to the problem formulation and paper writing. BH and GC developed the program for generating random graphs and testing the robustness of IPCA. The manuscript was written by ML. All authors read and approved the final manuscript.

## Supplementary Material

Additional file 1 **P-values for the predicted clusters with *size *≥ 6 generated using *T*_*in *_= 0.9 and *T*_*in *_= 0.6.** The data provided represent the statistical analysis of the predicted clusters. P-value is calculated for each predicted cluster and a function category is assigned to it when the minimum P-value occurs. When *T*_*in *_= 0.9, there are 132 clusters (*size *≥ 6) generated by IPCA. When *T*_*in *_= 0.6, there are 443 clusters (*size *≥ 6) generated by IPCA. Click here for file

Additional file 2 **Functional annotation for a predicted cluster of 10 proteins.** This file provides a cluster which is composed of ten proteins: YGL173c, YOL149w, YBL026w, YCR077c, YJR022w, YER112w, YER146w, YDR378c, YNL147w, and YLR438c-a. The functional annotations for each protein in the cluster are listed in this file. Click here for file
