# Supplementary material for: Modifying the DPClus algorithm for identifying protein complexes based on new topological structures
Source: BMC Bioinformatics. 2008 Sep 25;9:398. doi: 10.1186/1471-2105-9-398 (PMC2570695; doi:10.1186/1471-2105-9-398)
Supplement: Additional file 2 — Functional annotation for a predicted cluster of 10 proteins. This file provides a cluster which is composed of ten proteins: YGL173c, YOL149w, YBL026w, YCR077c, YJR022w, YER112w, YER146w, YDR378c, YNL147w, and YLR438c-a. The functional annotations for each protein in the cluster are listed in this file. [file 1471-2105-9-398-S2.doc]

Additional file 2: Functional annotation for a predicted cluster of 10 proteins

| ORF | Protein functional categories | | | | | | | | | | | |
| --- | --- | --- | --- | --- | --- | --- | --- | --- | --- | --- | --- | --- |
| YGL173c | 01.03.16.01 | 01.03.16.03 |  | 10.01.05 | 10.03.01 |  | 11.04.01 | 11.04.03 |  |  |  | 43.01.03.05 |
| YOL149w | 01.03.16.01 |  |  |  |  |  |  | 11.04.03 |  | 16.03.03 |  |  |
| YBL026w |  |  |  |  |  |  | 11.04.01 |  | 11.04.03.01 |  | 12.07 |  |
| YCR077c |  |  | 10.01.03 | 10.01.05 |  | 10.03.04.05 |  | 11.04.03 |  |  |  |  |
| YJR022w |  |  |  |  |  |  | 11.04.01 |  | 11.04.03.01 |  |  |  |
| YER112w |  |  |  |  |  |  | 11.04.01 |  | 11.04.03.01 |  |  |  |
| YER146w |  |  |  |  |  |  | 11.04.01 |  | 11.04.03.01 |  |  |  |
| YDR378c |  |  |  |  |  |  |  |  | 11.04.03.01 |  |  |  |
| YNL147w | 01.03.16.01 |  |  |  |  |  |  |  | 11.04.03.01 | 16.03.03 |  |  |
| YLR438c-a |  |  |  |  |  |  | 11.04.01 |  | 11.04.03.01 | 16.03.03 |  |  |

The functional category for each code listed in the table:

01.03.16.01: RNA degradation;

01.03.16.03: DNA degradation;

10.01.03: DNA synthesis and replication;

10.01.05: DNA recombination and DNArepair;

10.03.01: mitotic cell cycle and cell cycle control;

10.03.04.05: chromosome segregation/division;

11.04.01: rRNA processing;

11.04.03: mRNA processing (splicing, 5'-, 3'-end processing);

11.04.03.01:splicing;

16.03.03: RNA binding;

12.07: translational control;

43.01.03.05: budding, cell polarity and filament formation.
